# The impact of hemispherically asymmetrical volcanic aerosol injections on the North Atlantic Oscillation

**DOI:** 10.1038/s41598-025-16232-w

**Published:** 2025-08-26

**Authors:** Olivia T. Woods, James U. L. Baldini

**Affiliations:** Department of Earth Sciences, Durham University, Durham, DH1 3LE UK

**Keywords:** North Atlantic Oscillation, Volcanism, Weather, Climate, Aerosols, Sulphur, Atmospheric dynamics, Atmospheric science, Climate change, Palaeoclimate

## Abstract

Volcanic eruptions exert a considerable influence on global climate and may impact large scale modes of atmospheric variability, including the North Atlantic Oscillation (NAO). However, little consensus on the nature of this relationship exists, partially because of the numerous possible NAO forcing mechanisms, the large number of variables associated with eruptions (e.g., size, time of year, latitude, etc), the small number of large eruptions across the instrumental era, and conflicting climate model simulations. This study leverages statistical techniques to evaluate the relationship between historical volcanic eruptions and the observed NAO response since the beginning of the twentieth century. Monte Carlo analyses show that large northern hemisphere eruptions (VEI 5 or greater) are significantly related to a positive NAO response at the 1% significance level across the twentieth century, whereas no significant association is found for southern hemisphere eruptions, though this may reflect a low sample size. Furthermore, results suggest an increasing NAO response amplitude to northern hemisphere eruptions across recent decades, possibly illustrating dynamic changes in the atmospheric phenomenon resulting from anthropogenic activity.

**Supplementary Information:**

The online version contains supplementary material available at 10.1038/s41598-025-16232-w.

## Introduction

The North Atlantic Oscillation (NAO) is the principal mode of wintertime atmospheric variability in the North Atlantic basin, exerting a profound influence on temperature and precipitation patterns across Europe^1^. The NAO describes atmospheric pressure fluctuations between the Icelandic Low and the Azores High, where the NAO’s overarching climate impacts are well established: positive phases are typically characterised by mild, wet winters across northern and central Europe, whereas the opposite conditions prevail during NAO negatives. However, the root causes of anomalously strong or prolonged NAO phases, which have increased markedly over recent decades, are less well understood. These extreme NAO phases often result in severe storms and winter flooding^2^, which have important socio-economic and environmental implications for European countries. A more comprehensive understanding of NAO forcing mechanisms is therefore required to improve prediction and mitigation.

Whereas various external factors may impact the NAO, this study specifically focuses on the influence of volcanic eruptions. Despite being proposed over three decades ago^3^, the volcanism-NAO relationship remains controversial and poorly understood. The study aims to disentangle the volcanic signal from long-term NAO trends, providing clearer insights into specific atmospheric responses and potential causal mechanisms. The investigation spans the period from 1900 to 2022 CE and uses meteorological data, thereby minimising uncertainty. Statistical methods are employed to assess the plausibility of the volcanism-NAO relationship, complementing the array of modelling studies in existing literature [e.g.,^4,5^]. Importantly, here we focus on the impact of extratropical, rather than low latitude, eruptions on the NAO, because existing evidence suggests that extratropical eruptions may exert a considerable control on atmospheric circulation^6^.

## Background

### The North Atlantic Oscillation

The NAO is defined by sea level pressure (SLP) differences between the Icelandic Low (IL) and the Azores High (AH), reflecting atmospheric mass distributions from the Arctic to the subtropical Atlantic^1,7^. It is one of the most prominent large-scale modes in the Northern Hemisphere extratropics^8^. These pressure patterns significantly influence NH climate by affecting the strength and direction of westerly winds across the North Atlantic, which modulate heat and moisture transport to Europe^9^. Despite extensive study since the nineteenth century, understanding NAO amplitude, climatic modulators, and predictability remains challenging^10,11^. Even the latest climate models struggle to accurately capture long-term NAO variability^12^. This difficulty is often attributed to the northern polar atmosphere’s large internal variability and the vast range of parameters (e.g., Quasi-Biennial Oscillation and the El Niño–Southern Oscillation states, ozone and water vapor concentrations, feedback mechanisms, etc) that must be constrained^13^, underscoring the need for a deeper understanding of NAO modulating factors.

### Volcanic eruptions and climate

Volcanic eruptions can lead to a wide range of climate impacts, extensively summarized by Marshall et al.^14^. Stratospheric sulphate aerosols, formed from the reaction of injected volcanogenic sulphur compounds (e.g. SO_2,_ H_2_S) with atmospheric water following oxidation^15^, are the main drivers of climate variability following an eruption^16^. Unlike in the troposphere, where they are rapidly removed, stratospheric aerosols can persist for 1-3 years^17^. They exert a strong radiative impact by reflecting shortwave radiation back into space and absorbing both solar and terrestrial longwave radiation^18^. This results in a net negative perturbation to Earth’s radiation budget, typically causing surface cooling that peaks about one year after an eruption. Conversely, the lower stratosphere warms due to the absorption of longwave radiation sulphate aerosols^16^.

Some previous studies suggest that tropical eruptions induce a stronger global temperature response than mid-to-high latitude eruptions, where aerosols have a comparatively shorter-residence time and are confined to the eruption hemisphere^19^. Because most large eruptions observed since the beginning of the satellite era have been tropical, research has primarily focused on these events, whereas extratropical eruptions have received comparatively little attention. Despite recent research strongly suggesting that high-latitude NH eruptions have a much larger impact than previously thought^6^, considerable uncertainty remains regarding latitudinal effects on the climate response. As an example relevant to the NAO, the Intertropical Convergence Zone (ITCZ) moves away from the cooling hemisphere of the eruption to restore energetic equilibrium to the atmospheric system^20,21^. Because the ITCZ and the Azores High (AH) constitute the respective rising and falling limbs of the NH Hadley cell, the ITCZ’s position influences the size and location of the AH^22^, making it an important modulating factor of the post-eruptive NAO response. The precise details of how this process operates remain poorly understood.

### The Impact of volcanogenic aerosols on anthropogenically forced climate

Global warming trends and increasing background levels of stratospheric aerosols ultimately derived from human industrial activity contribute significantly to the uncertainty in predicting future changes in atmospheric dynamics^18,23^, including the NAO. Like volcanic aerosols, anthropogenic sulphate aerosols primarily influence climate via the scattering or reflection of incoming solar radiation. Focusing on the impact of anthropogenic aerosols on North Atlantic climate, Fischer-Bruns et al.^24^ projected an increase in extreme positive NAO responses and a decrease in extreme negative responses. Fuentes-Franco et al.^25^ suggest a significant weakening of NAO variability in the second half of the twenty-first century would occur under a high greenhouse gas emissions scenario (SSP5–8.5) in CMIP6 models, attributed to less intense negative NAO phases. Though not directly referencing the NAO, other studies suggest further possible influences^26^. These include an unprecedented expansion of the Azores High since 1850 relative to the past millennium, which has been linked to anthropogenic climate change^27^.

Additionally, Aubry et al.^17^ show that the climate response to future volcanism will vary depending on eruption magnitude. Moderate magnitude eruptions are expected to contribute 75% less stratospheric aerosol input due to the rising height of the tropopause under a high greenhouse gas emission scenario. Conversely, large eruptions (> VEI 5) are predicted to input proportionately more sulphur into the stratosphere due to decreasing aerosol particle size and an acceleration of Brewer-Dobson circulation. This is expected to exacerbate global-mean radiative forcing and stratospheric warming by 30% and 52%, respectively^28^.

### Proposed mechanisms for an NAO-volcanism relationship

Robock and Mao^3^ suggested a link between volcanism and the NAO existed when they identified anomalous NH winter warming within two years of major eruptions. Numerous studies have investigated this response, proposing that the observed temperature and SLP patterns align with a positive NAO phase^8^. Such studies have also frequently found stronger and more statistically significant NAO responses to tropical rather than extratropical eruptions^29^.

The most common mechanism explaining the positive NAO response to tropical volcanism involves the aerosol-induced perturbation of the meridional temperature gradient. Robock^16^ explains that low-latitude heating of the lower stratosphere following a tropical eruption enhances the pole-to-equator temperature gradient, strengthening the stratospheric polar vortex and enhancing westerly winds. These strong westerly winds prevent planetary waves from passing through and entering the upper stratosphere, instead reflecting them back down into the troposphere, creating a circulation pattern resembling a positive NAO. With fewer waves reaching the upper stratosphere, the risk of sudden stratospheric warming decreases, sustaining the polar vortex’s strong state throughout winter and resulting in an extended positive NAO signal. Another suggestion by Stenchikov et al.^30^ involves a reduced overall upward planetary wave flux due to the reduced tropospheric meridional temperature gradient following a tropical eruption, though Graf et al.^31^ later challenged this result.

There is little consensus across models on whether a relationship between extratropical volcanism and the NAO exists, so researchers have yet to propose a robust mechanism linking the two^5^. Surprisingly, minimal research considers the impacts of post-eruptive ITCZ migration on the NAO, despite their probable link and extensive study of the volcanism-ITCZ relationship^20,26^. Some studies link extratropical volcanism to a negative NAO state in the first two winters following an eruption^32^, others as a negative NAO state after the second year (followed by a positive NAO in years 3-4)^33^, and across both winter and summer^34^, but other models do not simulate this response^5,29^. Swingedouw et al.^35^ provide an excellent review on how eruptions affect major modes of atmospheric circulation, and report that across both the instrumental and past millennium, eruptions have tended to produce an NAO positive response up to four years after the eruption. An outstanding question is whether enough radiation reaches high latitudes during NH winter to cause the necessary radiative perturbation, especially given the high level of natural variability at high northern latitudes. Furthermore, Driscoll et al.^11^ found that Coupled Model Intercomparison Project 5 (CMIP5) models generally fail to capture the atmospheric NH response to volcanic eruptions. They suggest that these models frequently underestimate polar vortex strength, the NAO response, and associated temperature anomalies compared to observational data. However, other researchers observe significant polar vortex strengthening following an eruption using the same CMIP5 models and attribute less robust simulations to the inclusion of too many smaller magnitude eruptions^13^. The complexity of volcanic processes exacerbates these challenges, including the uncertain behaviour of volcanic aerosols^14^ and the modern deficit of large, well-studied eruptions. Polvani et al.^4^ have questioned whether the entire volcano-NAO relationship is even distinguishable from other natural variability, underscoring the need for an objective statistical evaluation, which this study aims to provide.

## Methodology

### Selection and manipulation of NAO timeseries

This study uses the NAO index updated from Jones et al.^36^, which records the difference in monthly normalised sea level pressure (SLP) between Gibraltar and southwest Iceland. Different indices were considered because the best locations for measuring the southern node vary seasonally due to the non-stationary nature of NAO pressure centres^37,38^. However, the Gibraltar record was selected because it makes a more suitable index pairing with Icelandic stations during winter^36^ and displays less inter-decadal variability compared to more western stations. This was deemed favourable for this study, because the relationship between individual eruptions and longer-term external trends are less likely to obscure the NAO signal.

**Fig. 1:**
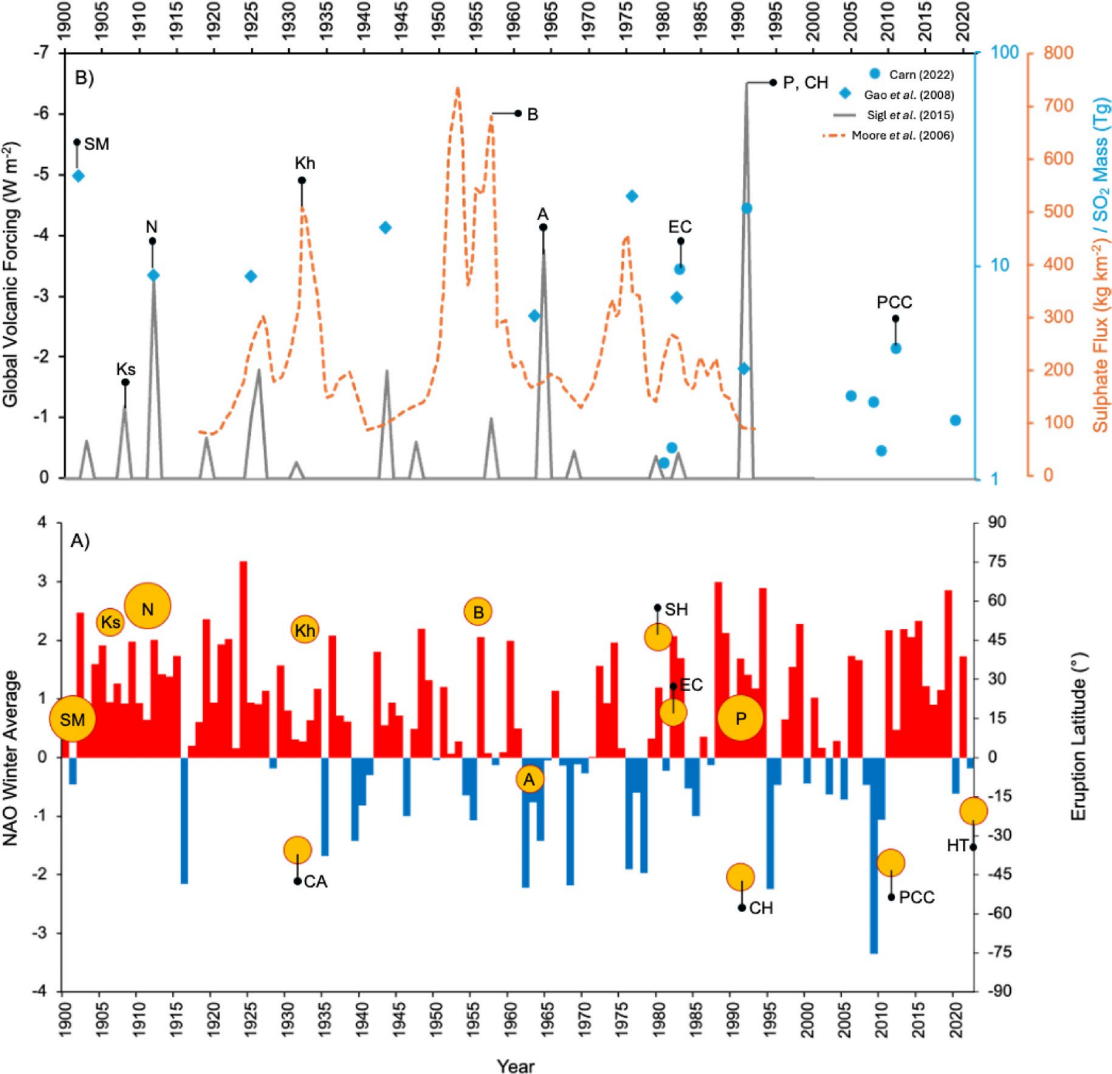
(**A**) Timeseries of mean DJF NAO indices for the period 1900–2022 (from https://crudata.uea.ac.uk/cru/data/nao/)^36^. Circles illustrate the timing and latitude of large (VEI5 or larger) eruptions within this window (see Table 2), where small and large circles represent VEI5 and 6 events respectively. (**B**) Global volcanic forcing (W/m^2^; 1900–2000 CE) derived from ice core sulphate deposition (^42^ , grey); yearly sulphate deposition (kg/km^2^; 1918–1996 CE) from an ice core in Lomonosovfonna, Svalbard (^51^, orange); global total stratospheric sulphate aerosol injection (Tg; 1900–2000 CE) (^52^, diamond blue markers); annual sulphur dioxide (SO_2_) emissions from 1979 to 2018 CE (Tg), determined from satellite measurements (^53^, circular blue markers). Peaks in these records corresponding to the eruptions identified in (A) have been denoted by matching black markers. Sulphate peaks for Cerro Azul (1932) and St Helens (1980) could not be identified.

Annual winter values were calculated as the December, January, and February (DJF) mean. Winter averages were dated with respect to the first month (December), rather than the last month (February), as is otherwise conventional (e.g., D1900, J1901, F1901, is referred to as winter 1900, rather than 1901). This approach was critical for the accuracy of the Monte Carlo analyses (as explained in Section "The Impact of volcanogenic aerosols on anthropogenically forced climate") and is maintained throughout the report for clarity. The NAO winter timeseries (Fig. 1) shows that positive winters were vastly more frequent over the study period than negative winters, which was considered when assessing the probability of a given response following an eruption. Significantly strong NAO positive and negative winter events (hereafter NAO^+^ and NAO^-^, respectively) were defined as being greater than one standard deviation above or below the long-term winter mean, to generate an appropriate number of events for Monte Carlo simulations (Table 1).

**Table 1:** NAO^+^ and NAO- events with their associated thresholds, used here to identify events.

NAO event and significance threshold	Years
Positive winters (>1.88)	1902, 1905, 1909, 1912, 1919, 1921, 1922, 1924, 1936, 1948, 1956, 1960, 1974, 1982, 1988, 1989, 1994, 1999, 2011, 2013, 2014, 2015, 2019
Negative winters (<− 0.66)	1916, 1935, 1939, 1940, 1946, 1955, 1962, 1963, 1964, 1968, 1976, 1978, 1985, 1995, 2005, 2009, 2010

### Eruption data and supplementary volcanic datasets

The eruption data were sourced from the Smithsonian Institution’s Volcanoes of the World database^39^. Eruption size was assessed based on the Volcanic Explosivity Index (VEI), with a focus on large eruptions defined as VEI 5 and above. Compared to previous centuries, volcanic activity since 1900 has been relatively subdued, with only 13 VEI 5 or above eruptions occurring during this period (Fig. 1). However, this study window was chosen to maximize the constraints on eruption and NAO event timing, thereby reducing uncertainty in the results. Accurate dating of eruptions was critical for this study and all start dates from the database were cross-referenced with existing literature (Table 2). For prolonged eruptions where the start date did not correspond to the date associated with the maximum VEI, the latter was used in statistical analysis. Sulphur emissions from the eruptions were also considered (Fig. 1), as well as the eruption timing.

**Table 2:** Geographical information of VEI 5 and 6 eruptions occurring since 1900.

Volcano	Latitude	Location	Eruption Date	Date of Max VEI	VEI	References
Santa María	14.75 °N	Guatemala	25/10/1902	–	6	Williams and Self (1983)
Ksudach	51.80 °N	Russia	28/03/1907	–	5	Macías and Sheridan (1995)
Novarupta	58.23 °N	AK, USA	06/06/1912	–	6	Hildreth and Fierstien (2012)
Cerro Azul	35.66 °S	Chile	–/–/1916	10/04/1932	5	Hildreth and Drake (1992)
Kharimkotan	49.12 °N	Russia	08/01/1933	–	5	Zuev et al. (2021)
Bezymianny	55.98 °N	Russia	22/10/1955	30/03/1956	5	Belousov (1996)
Mt. Agung	8.34 °S	Indonesia	18/02/1963	17/03/1963	5	Zen and Hadikusumo (1964)
Mt. St Helens	46.19 °N	WA, USA	27/03/1980	18/05/1980	5	Fruchter et al. (1980)
El Chichón	17.36 °N	Mexico	28/03/1982	04/04/1982	5	Varekamp et al. (1984)
Cerro Hudson	45.90 °S	Chile	08/08/1991	–	5	Naranjo and Stern (1998)
Mt. Pinatubo	15.14 °N	Philippenes	02/04/1991	15/06/1991	6	Westrich and Gerlach (1992)
Puyehue-Cordon Caulle	40.58 °S	Chile	04/06/2011	–	5	Elissondo et al. (2016)
Hunga Tonga-Hunga Ha’apai	20.55 °S	Tonga Islands	20/12/2021	15/01/2022	5	Gupta et al. (2022)

Due to the relatively low frequency of large volcanic eruptions from 1900 to 2022 CE, there were sometimes insufficient large eruptions to conduct robust statistical tests when additional controls such as eruption latitude were imposed. To address this, a scoring system was devised based on the broadly logarithmic nature of the VEI scale (VEI 1 = 1 point, VEI 2 = 10 points, VEI 3 = 100 points,..., VEI 6 = 100,000 points). This scoring system allowed for the calculation of an overall score for each year based on total volcanic activity (Table 3). Separate lists were created for Northern Hemisphere (NH) and Southern Hemisphere (SH) eruptions, from which the 15 highest scoring years were selected for Monte Carlo analysis if the number of VEI 5 and 6 eruptions was insufficient for robust analyses. These datasets are referred to as ‘points determined years’ henceforth.

**Table 3:** Top 15 highest scoring years of volcanic activity in the northern hemisphere (A) and the southern hemisphere (B). Scoring accounted for all eruptions in the given year and was weighted based on VEI, with VEI 1 = 1 point, VEI 2 = 10 points, VEI 3 = 100 points,..., VEI 6 = 100,000 points. Note that years post-2019 was substituted for the next highest scoring year during NAO^+^ simulations due to the absence of a postdating NAO^+^ event: 2020 (NH) and 2021 (SH) were replaced with the years 1917 (1392 points) and 1994 (1169 points) respectively. A further 6 substitutions were required in NAO- analyses, due to the absence of a NAO- even since 2010; the years substituted in were 1953, 1974, 1947 (NH) and 2008, 1982, 1990 (SH).

(A)	
Year	Total points (NH)
1902	102,403
1991	100,413
1912	100,341
1933	11,192
1980	10,559
1956	10,286
1982	10,277
1907	10,110
1986	3,515
2008	2,566
1981	2,447
2020	2,438
1924	2,321
2011	2,295
1931	2,294
2013	1,685
2010	1,659
2015	1,600
1929	1,444
1999	1,397

(B)	
Year	Total points (SH)
1932	10,452
1963	10,320
1991	10,175
2011	10,075
2021	10,035
2014	3,436
1951	2,224
1919	2,050
2010	1,442
2017	1,337
1955	1,321
2002	1,306
1966	1,264
1911	1,261
1937	1,220
1968	1,213
2000	1,204
1904	1,201
1933	1,201
2006	1,177

VEI is a generally reliable indicator of an eruption’s explosivity and size. However, the scale has some notable limitations, which occasionally make VEI scores misleadingly high, particularly when used as an indicator of climatic impact^40^. Therefore, two records of stratospheric aerosol optical depth at both global and inter-hemispheric scales (updated from Sato et al.^41^ and Sigl et al.^42^) were also analysed for comparison. The spikes in the records, indicative of volcanic radiative forcing, were identified and the corresponding dates were treated akin to the eruption years. However, such analyses proved problematic: a common issue was that peak AOD was frequently not reached until the year following a known eruption, and therefore when these dates were input into the simulation, eruptions where a winter NAO event began in the same year as the eruption were missed in the selection process. A threshold-based approach was also trialled for selecting years, but consequently AOD exceeded the boundary for several years during prolonged eruptions, resulting in several inputs relating to the same event. Therefore, the use of confirmed eruption dates rather than AOD estimates or ice core sulphur concentrations was deemed most appropriate for the analyses.

### Monte Carlo analysis

The probability of NAO event timing being associated with major volcanic eruptions was assessed using Monte Carlo analysis in MATLAB (see Supplementary Materials). To summarize the timing relationships between individual eruption years and their closest NAO positive or negative event (defined here as greater than one standard deviation above or below the long-term winter mean), the root-mean-square (RMS) statistic was calculated^21^. The RMS statistic essentially quantifies the differences between one set of values and a second set. The RMS value derived from the actual eruption and NAO datasets was compared against an RMS probability distribution generated by 1 million randomly simulated datasets of NAO event dates, while keeping eruption dates fixed. The p-value was calculated as the percentage of simulated values with an RMS less than or equal to the observed value (i.e., the simulated NAO event dates which were closer to the fixed eruption dates than the actual NAO event dates), indicating the likelihood that the two datasets were temporally independent. A significance level of 5% (p = 0.05) was used, reflecting 95% confidence that the observed RMS statistic did not happen by chance. The lower the p-value, the lower the probability that the observed RMS statistic occurred by chance.

For the study period covering 1900-2022 CE, the dates of all large eruptions and NAO events are well constrained, and the RMS calculation only considered positive date differences, where the NAO event occurred after the eruption. It was crucial to associate the winter NAO values with the year’s end, rather than the beginning, to ensure that NAO events never preceded eruptions when exact date matches were found. Eruptions occurring too recently to have a reasonably long NAO record following them (e.g. Hunga Tonga, 2021) were omitted from the analyses due to lack of sufficient record length for the calculations of the RMS values. Similarly, in the case of ‘points determined years’ experiments, dates that could not be run due to the absence of a postdating NAO event were instead substituted with the next highest scoring year.

### Reanalysis data

Mean sea level pressure (SLP) was plotted for winters following all large eruptions occurring since 1900 CE using the twentieth century Reanalysis Monthly Composite tool from the NOAA Physical Sciences Laboratory. These SLP plots were utilized to assess the size and position of the Azores High (AH) and Icelandic Low (IL), providing insights into NAO state and subsequent climatic conditions over Europe following each eruption. Randomly selected years were also plotted for comparative analysis. In addition to examining SLP patterns, anomalies were analysed to quantify the frequency of observed post-eruptive responses relative to all winters from 1806 to 2015 CE.

## Results and discussion

### Initial Monte Carlo analyses

#### NAO positives

**Fig. 2:**
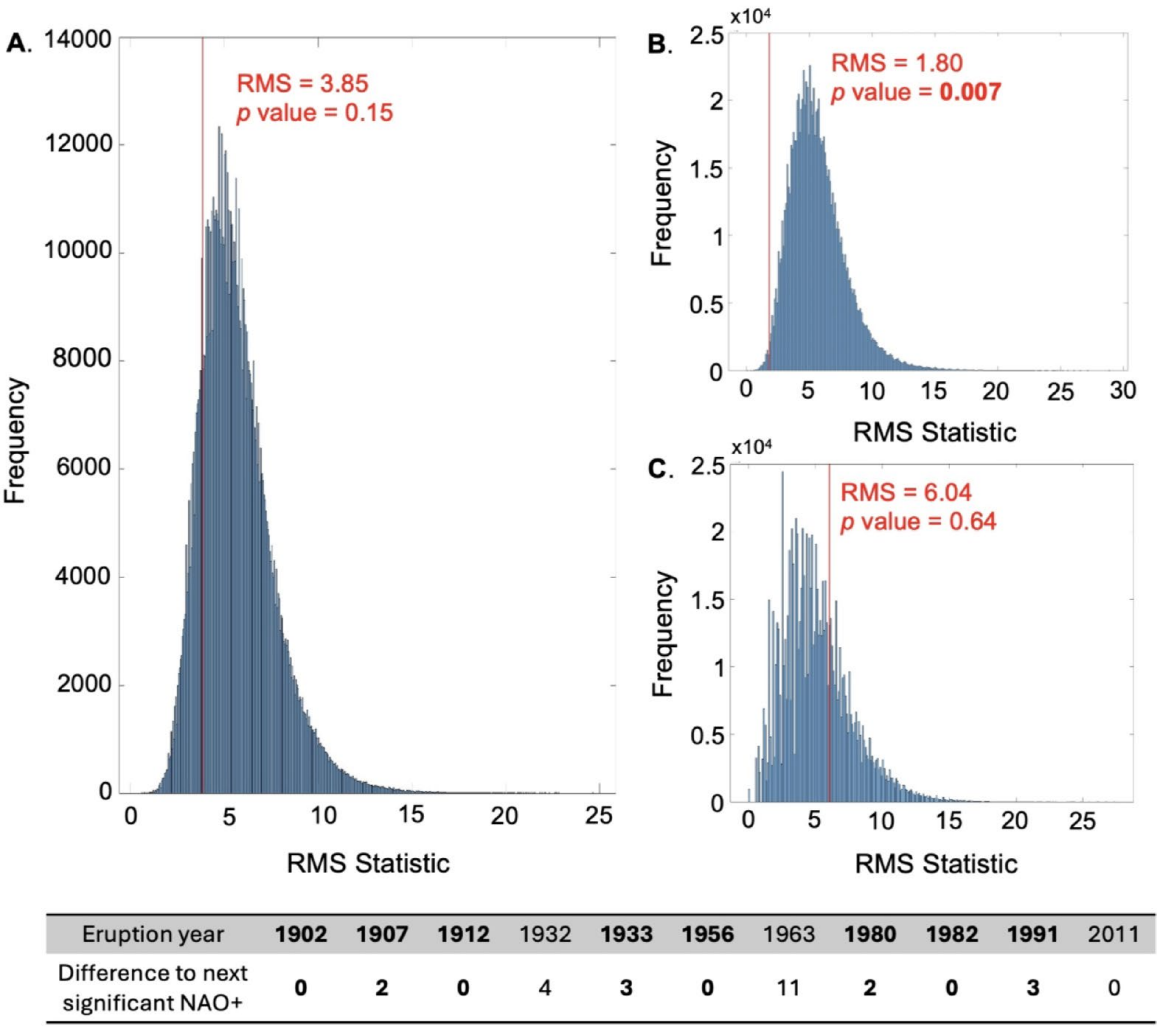
Monte Carlo simulations of global (**A**), northern hemisphere (**B**) and southern hemisphere (**C**) VEI 5 and 6 eruptions against all significantly positive NAO winters (>1.88). The RMS probability distribution generated from randomly simulated surrogate NAO datasets is shown in blue. The relative position of the true RMS value is denoted by the red line and labelled with the associated p value. The underlying table contains the calculated differences between each eruption and the NAO^+^ event (RMS calculation inputs). NH values are shown in bold. Note that 1991 CE is included in both NH and SH simulations due to the aerosols affecting both hemispheres.

An initial Monte Carlo simulation integrating all VEI 5 and 6 eruption years since 1900 CE, regardless of their latitude, against NAO^+^ events (Fig. 2A) reveals no significant relationship between the datasets (*p* > 0.05). However, upon distinguishing between hemispheres, significant results emerge, showing a pronounced association between VEI 5 and 6 NH eruptions and NAO^+^ events (*p* < 0.01) (Fig. 2B). Specifically, a significantly positive NAO^+^ event has occurred within three years of every major NH eruption since 1900. This timeframe is consistent with the persistence of volcanic aerosols in the stratosphere, which can extend up to three years post-eruption^16,18^ but depends on the eruption latitude^6^. Excluding Mt. St Helens (1980) from NH analyses had no discernible impact on results, despite the eruptions’ notably low sulphur content, and results remained significant at the 1% significance level (*p* = 0.009).

We then tested the relationship using the 15 highest points-determined years of NH volcanism (Table 3), which reinforces this correlation (*p* = 0.002), suggesting that the NAO is sensitive to not only large VEI 5 and 6 eruptions but also to more frequent VEI 4 NH eruptions. Conversely, SH VEI 5 and 6 eruptions show no discernible relationship with NAO^+^ events (*p* = 0.64), though the low eruption number limits the analysis’ robustness (Fig. 2C). Even when analysing the highest 15 points-determined years of SH volcanic activity, no significant relationship is found (p = 0.43).

#### NAO negatives

Identical analyses to those described above were conducted for NAO- events, but no significant relationship was found for either global or major NH eruptions, yielding p values of 0.31 and 0.43, respectively. The analysis for major SH eruptions could not proceed due to the limited number of suitable dates, exacerbated by the absence of significant NAO- events since 2010, resulting in the exclusion of both the Hunga Tonga 2021 and Puyehue-Cordón Caulle 2011 eruptions from the analyses. When the highest 15 points-determined years of SH volcanic activity were considered instead, a slightly more significant correlation was observed (*p* = 0.17) compared to NH eruptions, although it did not reach the minimum significance threshold (*p* = 0.05).

These results align broadly with existing research, including other statistics-based studies [e.g.,^8^], which generally do not identify a link between negative phases of the NAO and volcanic activity. However, the weak correlation with NH eruptions observed here contrasts with findings from the modelling results of Gudlaugsdóttir et al.^33^, Zambri et al.^32^ and Sjolte et al.^34^, who found that large extratropical eruptions were associated with a negative NAO response. Isolating extratropical NH eruptions (>30° N) from the simulation did not notably affect the observed significance (*p* = 0.32), which is consistent with the robust relationship already identified for NAO^+^ events.

### Monthly-resolved NAO responses following NH eruptions

**Fig. 3:**
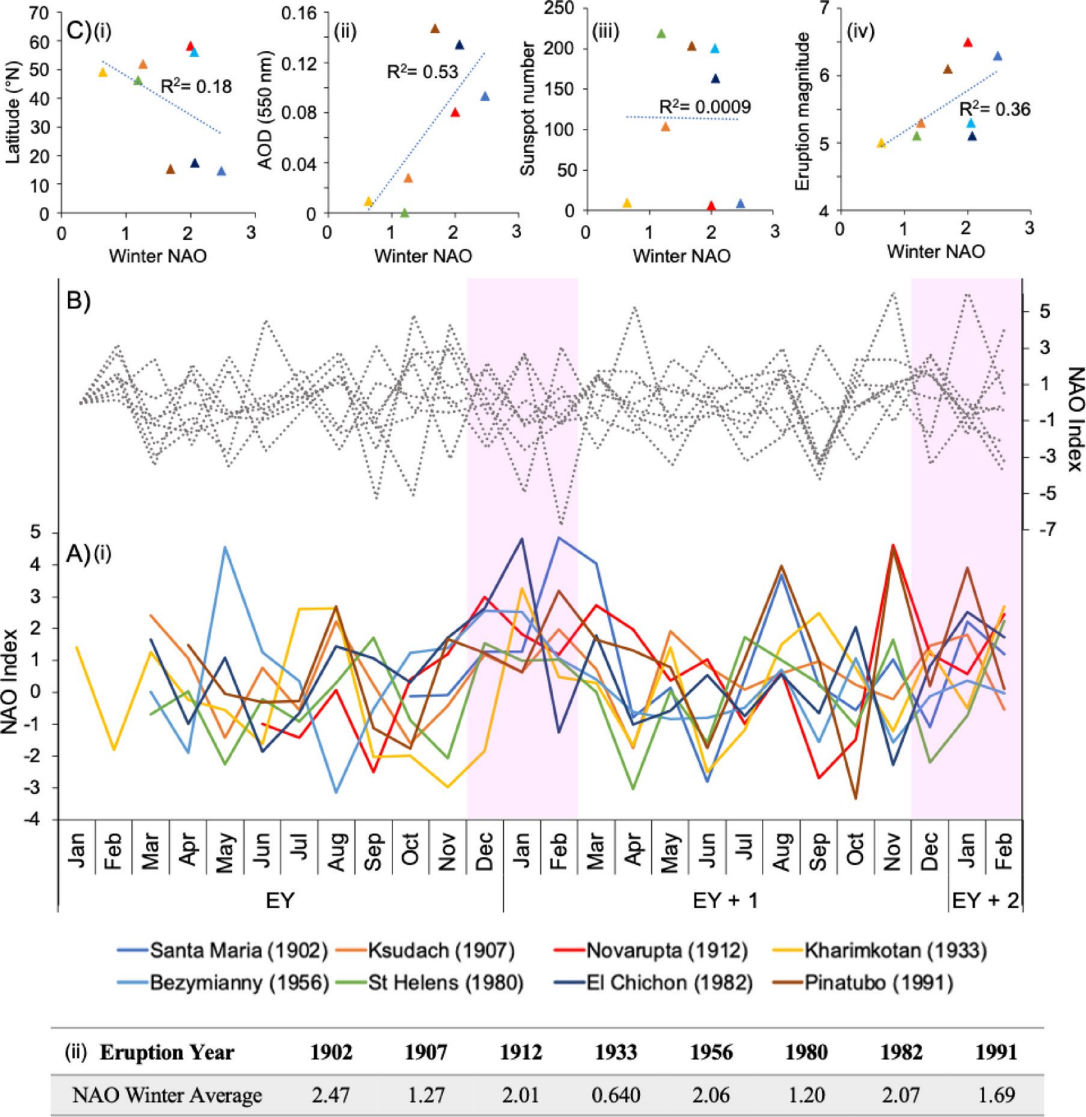
(**A**) Monthly NAO timeseries following all VEI 5 and 6 eruptions, which begin the month of the eruption and continue to the end of the second winter after the eruption year (EY). Shaded regions highlight DJF trends corresponding the averages presented in A(ii). (**B**) NAO timeseries of 8 randomly simulated non-volcanic years (1926, 1931, 1935, 1940, 1954, 1971, 1998, 2009). (**C**) Scatter correlations comparing the amplitude of the NAO response to the eruption latitude (i), stratospheric aerosol optical depth (AOD) (ii;^41^), sunspot number (iii; sourced from the Sunspot Index and Long-term Solar Observations database) and eruption magnitude (iv). Marker colours correspond to the legend in Part A. The coefficient of determination (R^2^) is also presented, however, note that the distributions are not normally distributed given the small number of datapoints and the R^2^ provided will not be statistically significant. The Sato et al.^41^ AOD record analysed in Fig. 6C(ii) does not record a peak for the 1956 Bezymianny eruption, contrary to estimates from other studies (e.g.,^42^), and therefore this data point is not included in the plot.

Following the identification of a significant relationship between major NH volcanism and NAO^+^ events, monthly NAO index values were plotted up to the end of the second winter following each eruption (Fig. 3). These plots revealed a noticeable upward anomaly in index values during the first winter, highlighting a consistent positive NAO signal post-eruption. In contrast, a comparison with eight randomly selected years not associated with major NH volcanism (Fig. 3) showed no similar trend, again consistent with a non-random NAO response after eruptions. Given the higher frequency of positive NAO winters compared to negative ones, the probability of randomly selecting eight positive years is relatively high (~6%). However, the probability that half of these winters exhibit statistically significant NAO^+^ events (>1.88) is less than 1% (*p* = 0.0025), strongly suggesting that NH volcanism pushes the NAO into a stronger positive state relative to background conditions. Conversely, the NAO response in the second winter appears more random, consistent with findings from other studies^8^.

Additionally, a moderate correlation (R^2^ = 0.53) exists between the NAO response amplitude and stratospheric aerosol optical depth. Interestingly, the mechanism proposed by Graf et al.^43^, which relies on the creation of an enhanced stratospheric temperature gradient, does not apply to high-latitude NH eruptions. This suggests that extratropical eruptions induce a different aerosol-mediated NAO response, which current models have not fully captured, potentially due to uncertainties in aerosol behaviour^14^ or limited observational constraints on large high-latitude eruption impacts in the satellite era^6^.

Furthermore, a moderate correlation exists between eruption magnitude and the NAO amplitude: the NAO response sensitivity to eruption magnitude supports the use of VEI as an appropriate measure of eruption size for this study (given a generally linear relationship between VEI and magnitude), despite potential limitations^40^. The impact of eruption season on the NAO was also considered, given its known influence on climate response to volcanic events^44^. However, due to the clustering of considered eruptions between March and June, conclusive inferences regarding seasonality effects could not be drawn from this dataset.

### Inferences from SLP reanalysis data

**Fig. 4:**
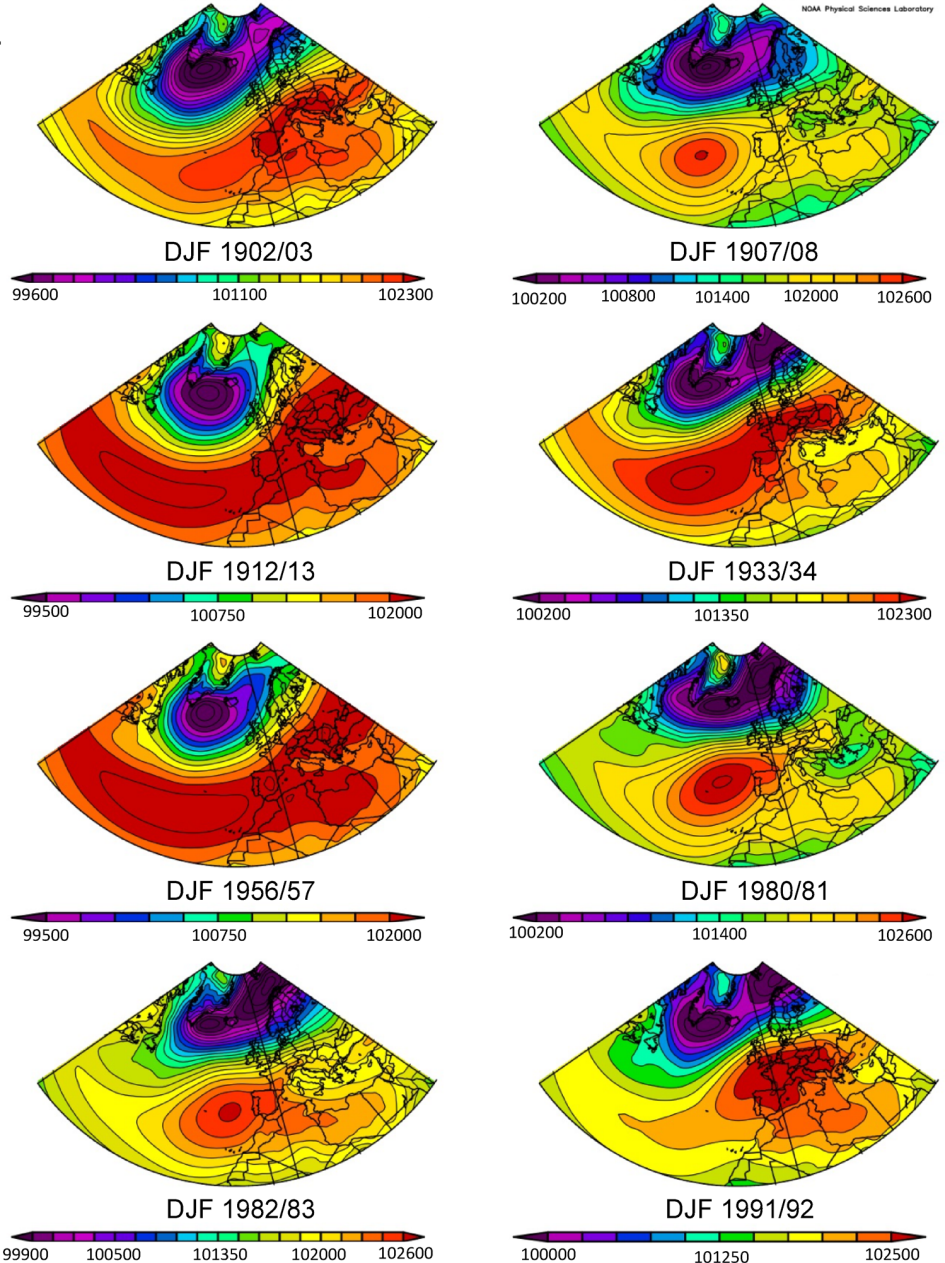
Mean sea level pressure (SLP) over the North Atlantic Region in winters following all major NH eruptions plots generated using NOAA Physical Sciences Laboratory twentieth century reanalysis data.

The analysis of mean sea level pressure (SLP) plots across Europe and the North Atlantic provides valuable insights into the behaviour of the Azores High (AH) and Icelandic Low (IL) pressure centres, shedding light on the NAO response following NH eruptions (Fig. 4). Using the NOAA tool, each plot’s unique scale initially portrays varying AH extents. However, when considering absolute SLP values, a consistent pattern emerges across the North Atlantic and western Europe during the winter following all eruptions, characterised by SLP values of at least 102,000 Pa. This threshold, identified in previous research by Davis et al.^45^, signifies an extensive AH presence typical of a positive NAO phase. Although some variability exists in the size and exact location of AH pressure maxima, indicated by the red zones on the map, the post-eruption pressure centre is often found over the Azores. A notable exception is observed following the Pinatubo eruption in 1991 CE, where the AH appears eastwardly displaced—a feature more commonly associated with a SH response and discussed further below.

The strength of the IL shows more variability between eruption years, with pressure minima over Iceland ranging from 995 to 1002 hPa. No clear relationship exists between the response and eruption latitude, with both the strongest and weakest IL occurrences corresponding to high-latitude eruptions, and with low latitude NH eruptions linked to intermediate IL strengths. However, winters with the weakest IL coincide with eruptions characterised by substantially lower stratospheric aerosol optical depth (AOD) (e.g., Ksudach 1907, Kharimkotan 1933, St Helens 1980) (Fig. 3), suggesting that polar vortex strength changes and dependence on stratospheric aerosol concentrations may influence the post-eruptive response at the northern node of the NAO.

**Fig. 5:**
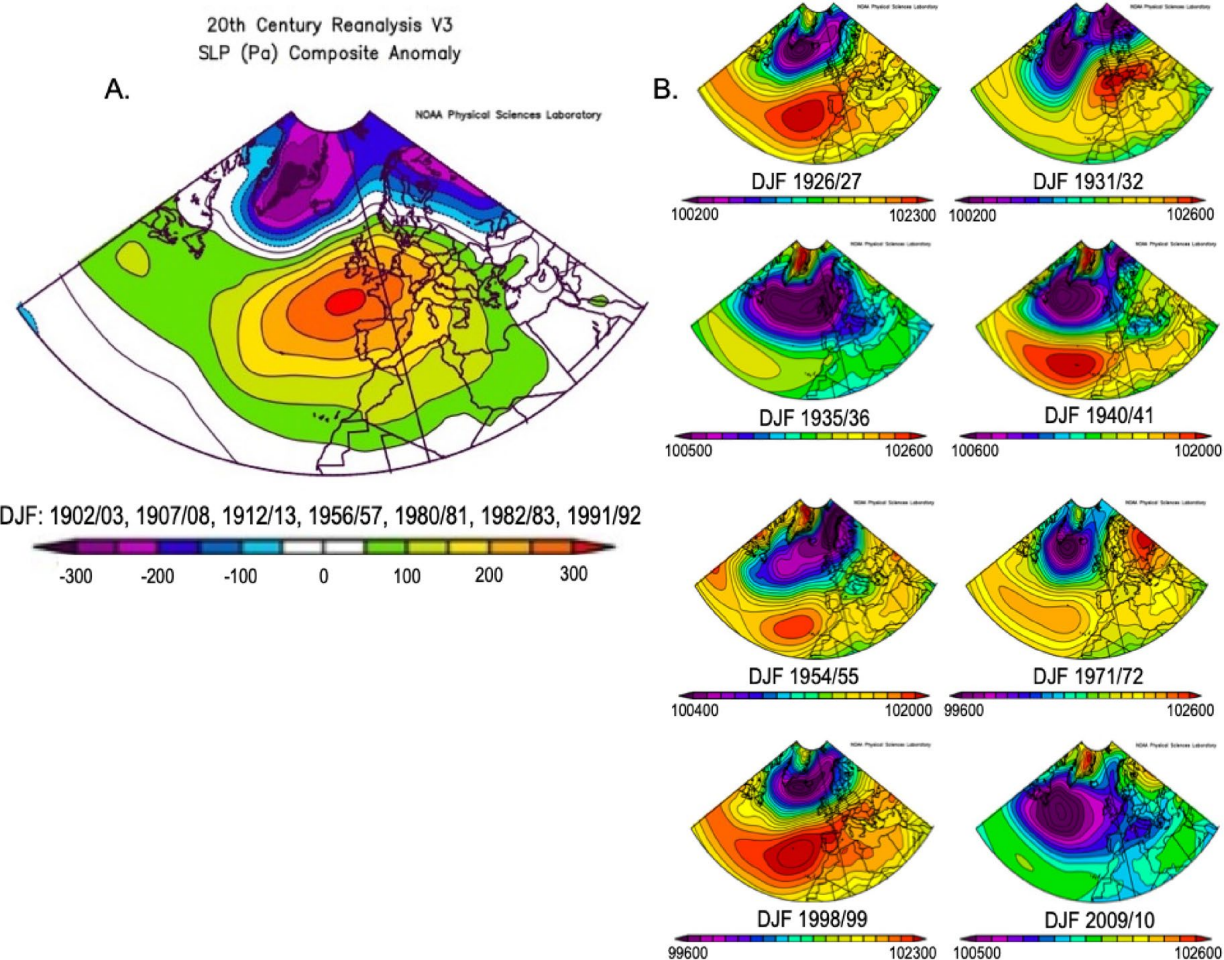
(**A**) North Atlantic SLP anomalies in post-eruptive winters, calculated across the period 1836–2015. (**B**) Mean SLP plots of eight randomly simulated non-volcanic years (1826, 1931, 1935, 1940, 1954, 1971, 1998, 2009).

Plots of eight randomly simulated years (Fig. 5) illustrate the significant variability in the extent and intensity of both pressure centres independent of volcanic activity. In contrast, post-eruption responses appear to exhibit a consistently uniform AH and IL configuration. The low-pressure region around the IL remains confined to high latitudes relative to random datasets, indicating a relatively strong jet stream and polar vortex inferred for all post-eruption winters, despite variations in SLP minima.

An anomaly plot of post-eruptive winters (Fig. 5) reveals that pressure centres are strengthened compared to the mean since 1836, underscoring that the NAO response to volcanic activity is notably stronger than the predominantly positive background conditions observed throughout the study period. Contours compressed at the northern end of the AH and the position of the strongest anomaly southwest of the UK suggest a relative northward AH shift in post-eruption winters compared to its mean position. This observation is consistent with previously observed and modelled results following eruptions like 1982 El Chichón and 1991 Pinatubo^43^, and this data suggests it applies to older and more northerly eruptions as well.

This northward shift could potentially be explained by the expansion of Hadley circulation, driven by factors such as a reduced tropospheric meridional temperature gradient or stratospheric ozone depletion, processes observed following volcanic aerosol influxes^16^. Given that ozone concentrations are highest towards the poles, high-latitude NH eruptions may have the greater influence via this mechanism, highlighting its importance for further study, especially because aerosol concentrations may influence the NH response but not align with the stratospheric gradient mechanism consistently.

### Possible modulators of NAO response amplitude

The study of the Arctic Oscillation (AO) has suggested that volcanic aerosol amounts directly reaching the polar vortex can significantly influence the AO response^46^. Although no similar mechanism has been proposed for the North Atlantic Oscillation (NAO), the strong link between these two modes of variability and the lack of a well-established NAO forcing mechanism for extratropical volcanism make this a plausible consideration for further investigation.

The climate impacts of volcanic eruptions occurring on the Kamchatka peninsula, including events like Ksudach 1907, Kharimkotan 1933, and Bezymianny 1956 CE, appear linked to polar vortex state preceding the eruption. For instance, Qu et al.^46^ demonstrate with the 1987 Shiveluch eruption that high-pressure ridges, located at the polar vortex gyre’s periphery and characterized by sinking, diverging air, can obstruct significant amounts of volcanic dust and aerosols from reaching the Arctic region. This suggests that pre-existing polar vortex state could explain why eruptions of similar magnitude, such as Ksudach 1907 and Bezymianny 1956, resulted in notably different NAO responses. Moreover, the 1991 Pinatubo eruption, despite being the strongest volcanic event of the twentieth century, exhibited a relatively muted response. Qu et al.^46^ propose that the low latitude of Pinatubo led to substantial aerosol loss within various atmospheric circulation belts before reaching the Arctic, thus reducing its influence on polar vortex strength and the eruption’s impact on the AO. However, this explanation does not align well with the findings of this study. For instance, other low-latitude eruptions like Santa María 1902 and El Chichón 1982 produced some of the largest winter NAO index values. This discrepancy suggests that factors beyond aerosol transport dynamics play a role in shaping the NAO response to volcanic eruptions.

Another factor influencing the subdued response of Pinatubo could be related to the more symmetrical hemispheric distribution of its aerosols compared to other NH eruptions^47^. Additionally, concurrent environmental factors such as the easterly phase of the Quasi-Biennial Oscillation (QBO), which tends to weaken the polar vortex and promote more negative NAO indices, may further bias the NAO towards a less positive response following the Pinatubo eruption. It is also possible that a subpolar and eastern tropical North Atlantic SST cooling leads an NAO positive event^48^. Gastineau and Frankignoul^48^ suggest that surface changes in the western subpolar region might modify transient and stationary eddies, resulting in a positive feedback leading to a large-scale equivalent barotropic signal. Volcanic aerosols may induce this initial SST cooling. Overall, whereas volcanic aerosol transport and aerosol interactions with atmospheric circulation patterns are crucial for understanding volcanic impacts on climate, the nuanced NAO responses following different eruptions highlight the need for continued research into the specific mechanisms driving these interactions.

### NAO response to SH eruptions

**Fig. 6:**
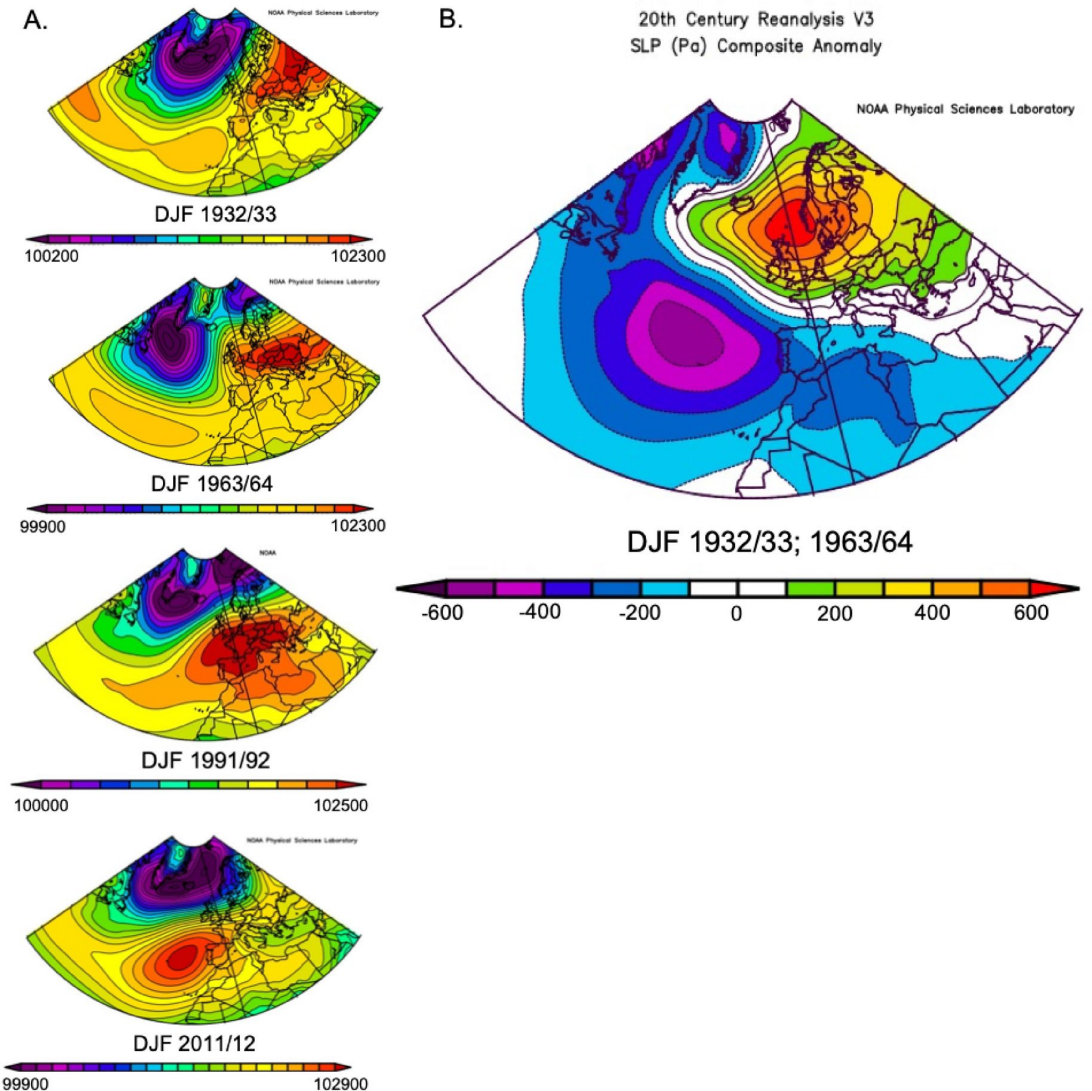
(**A**) Mean North Atlantic winter sea level pressure following southern hemisphere eruptions. (**B**) SLP anomaly plot following the eruptions of Cerro Azul 1932 and Mt Agung 1963 relative to the period 1836–2015.

Although we did not find any significant relationship between Southern Hemisphere (SH) eruptions and the North Atlantic Oscillation (NAO), reanalysis plots illustrate a distinct sea level pressure (SLP) configuration in winters following SH eruptions compared to those following Northern Hemisphere (NH) eruptions (Fig. 6). This configuration often shows a notable eastward displacement of the Azores High (AH). Although this trend also appears in the random dataset (indicating it is not entirely unique (e.g., 1931/32, 1971/72) (Fig. 5)), the presence of positive and negative anomalies up to 600 Pa following eruptions like Cerro Azul 1932 and Mt Agung 1963, where the eastward displacement is most pronounced, suggests this pattern is highly unusual (Fig. 6). A subtler shift occurred in winter 1991/92, associated with the VEI 5 eruption of Cerro Hudson in August 1991 and the hemispherically symmetrical aerosol distribution from Mt Pinatubo, which would have resulted in significant SH aerosol concentrations that winter.

Despite these observed patterns, there were too few twentieth century examples of large SH eruptions to statistically evaluate their relationship to the NAO with our techniques, making any identified similarities somewhat speculative. However, the occurrence of such a highly anomalous pattern in three out of four SH post-eruptive winters does warrant further research. Additionally, the SLP data highlight a limitation of this study: the use of a two-point station index to quantify the NAO. For example, strong similarities in dynamic responses in the winters of 1932/33 and 1963/64 exist, despite significantly different NAO index values (0.275 and -0.763 respectively^36^). This discrepancy is largely attributable to the inability of two-point station indices to account for dynamic shifts in the centres of action, as demonstrated here under extreme SLP configurations^49^.

### Changes across the study window and future trends

**Fig. 7:**
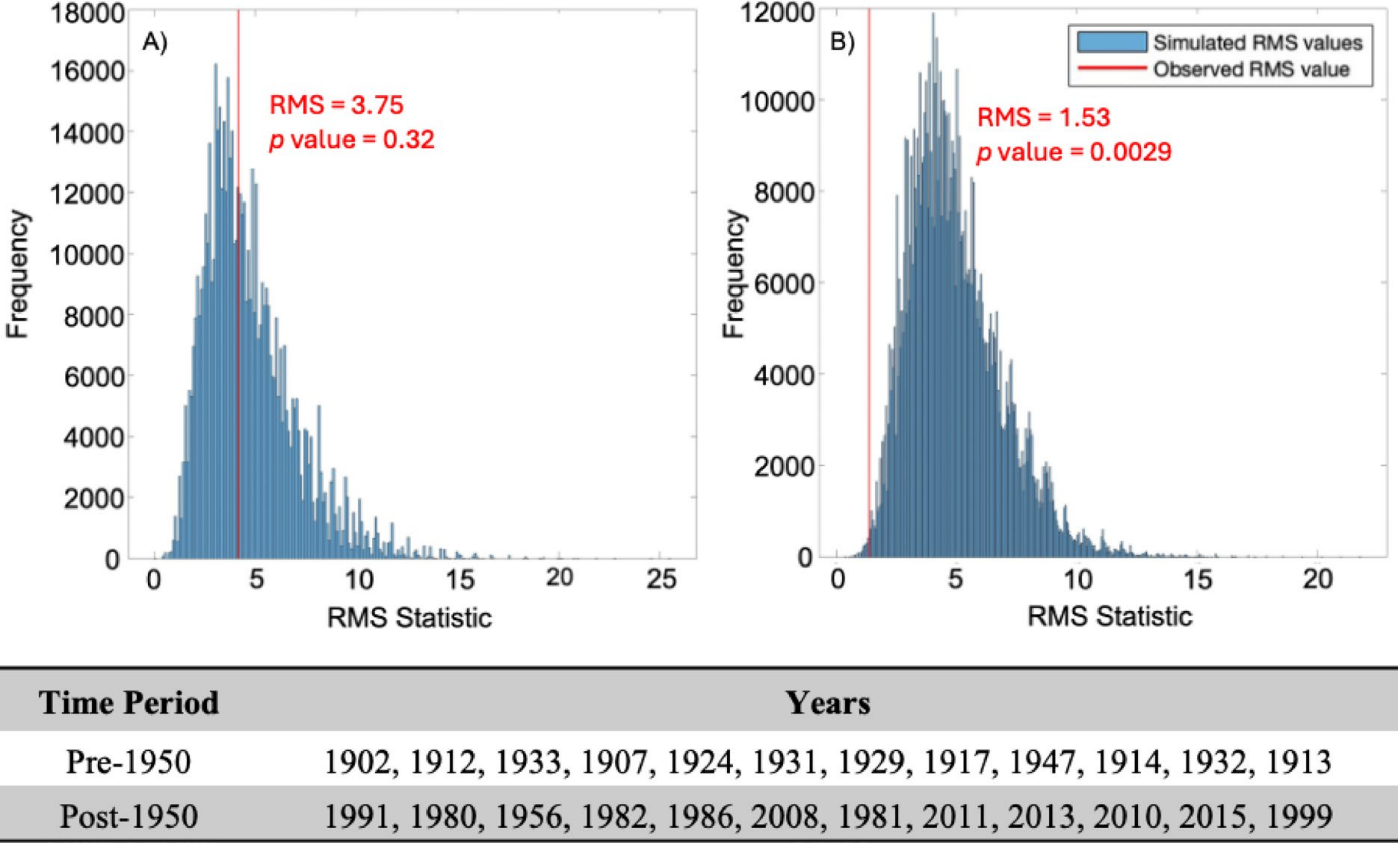
Monte Carlo analyses of points determined years of moderate volcanic activity (eruptions VEI 4 and below) against NAO^+^ events for the period 1900–1950 (A) and 1950–2022 (**B**). The years included in each simulation are given in the table, in the order of highest to lowest points scored.

Monte Carlo analyses were run pre- and post-1950 to investigate whether the acceleration of global industrialisation and anthropogenic emissions has influenced the NAO response to volcanism. Because the significance of major NH eruptions across the study window has already been identified, VEI 5 and 6 eruptions were omitted from these simulations, which were instead designed to see whether the NAO has become more-or-less sensitive to more moderate volcanic activity under increasing background concentrations of anthropogenic stratospheric sulphate aerosols. Monte Carlo analyses of the highest ‘points determined’ years, considering only eruptions VEI 4 and below against NAO^+^ events, strikingly demonstrate how pre-1950, moderate NH volcanic activity does not appear correlated to NAO^+^ events (*p* = 0.32), whereas post-1950 the twelve highest years of volcanic activity are correlated with an NAO^+^ at the 1% level (*p* = 0.0029) (Figure 7). This does not necessarily mean that the NAO was not sensitive to VEI 4 eruptions in the past but suggests that the sensitivity may have increased since 1950, meaning that the response associated with these eruptions more frequently surpasses the NAO threshold value considered in the Monte Carlo simulation (>1.88). These results seem consistent with other studies proposing that rising background aerosol concentrations are biasing the NAO towards more positive conditions^25^ and more frequent extreme positive events^24^. However, the possibility of the NAO becoming generally more sensitive to smaller volcanic sulphur injections under rising background concentrations may also be plausible, given that some studies suggest a threshold aerosol concentration is required to trigger the NAO response^50^. Finally, these results are important from a geoengineering perspective, where injection of sulphur into the NH to counteract global warming may result in a shift to a NAO^+^ phase.

## Conclusions

Monte Carlo analyses revealed that Northern Hemisphere (NH) eruptions are significantly related to positive phases of the NAO at the 1% level from 1900 to 2022 CE. All NH Volcanic Explosivity Index (VEI) 5 and 6 eruptions since the beginning of the twentieth century exhibited a sustained positive NAO signal in the first winter following the eruption. The amplitude of this positive response was generally well correlated with stratospheric aerosol optical depth (AOD), suggesting a mechanism mediated by atmospheric sulphate aerosol density driving the post-eruptive NAO response.

The NAO response to NH eruptions evolved across the twentieth century, suggesting an increase in the amplitude of the response from 1950 to the present day. These results align with the perspective that increasing anthropogenic sulphur dioxide and greenhouse gas emissions may be pushing the NAO towards more frequent and extreme positive conditions. Consequently, future NH volcanic sulphate contributions may cause a strongly positive NAO response. These results contribute to understanding how climate may respond following future volcanic eruptions, but at the same time highlight the need for further research to comprehensively understand the relationship between volcanic eruptions and the NAO.

## Supplementary Information

Below is the link to the electronic supplementary material.

Supplementary Material 1

## Data Availability

The datasets analysed during the current study are the updated NAO index data of Jones et al. (1997), which can be found at: https://crudata.uea.ac.uk/cru/data/nao/, and historical eruption data, which was sourced from the Smithsonian Institution’s Volcanoes of the World database: https://volcano.si.edu/#.
