# Supplementary material for: The impact of hemispherically asymmetrical volcanic aerosol injections on the North Atlantic Oscillation
Source: Sci Rep. 2025 Aug 26;15:31329. doi: 10.1038/s41598-025-16232-w (PMC12381042; doi:10.1038/s41598-025-16232-w)
Supplement: Supplementary file 1 — Supplementary Material 1 [file 41598_2025_16232_MOESM1_ESM.docx]

**Supplementary material for:**

**The impact of hemispherically asymmetrical volcanic aerosol injections on the North Atlantic Oscillation**

Olivia T. Woods^1^ and James U.L. Baldini^1*^

^1^Department of Earth Sciences, Durham University, Durham DH1 3LE, United Kingdom

*Corresponding author

**7) Appendices**

**Appendix 1** – Copy of MATLAB code

%Defining the study window and create an array of dates in prep. for MCS

study_window= [1900: 2022];

study_window_length = length(study_window);

% NAO and volcanic forcing datasets

% Input either volcanic forcing data/confirmed eruption years

%Positive NAO events defined as greater than 1 SD above the long term winter average

nao_years =[1902, 1905, 1909, 1912, 1919, 1921, 1922, 1924, 1936, 1948, 1956, 1960, 1974, 1982, 1988, 1989, 1994, 1999, 2011, 2013, 2014, 2015, 2019];

vol_forcing_years = [1902, 1907, 1912, 1932, 1933, 1956, 1963, 1980, 1982, 1991, 2011];

% Calculate the differences between the sets of dates

% Create a matrix to store the results of all volc years - all NAO years

differences_matrix = zeros(length(vol_forcing_years), length(nao_years));

% Perform subtraction and store the results in the matrix

for i = 1:length(vol_forcing_years)

for j = 1:length(nao_years)

differences_matrix(i, j) = nao_years(j) - vol_forcing_years(i);

end

end

% Display the result matrix

%columns are NAO years (from oldest to most recent) and rows

%are volcanic forcing years (oldest to recent)

disp('Result Matrix (NAO years - Volcanic Forcing Years):');

disp(differences_matrix);

% Find the smallest positive value in each column of the matrix

%Create an empty array to store the smallest positive value in each column

smallest_positive_differences = zeros(1, size(differences_matrix, 1));

for row = 1:size(differences_matrix, 1)

row_values = differences_matrix(row, :); % Extract column values

positive_values = row_values(row_values >= 0); % Filter positive values

if ~isempty(positive_values)

smallest_positive_differences(row) = min(positive_values); % Find the smallest positive value

else

smallest_positive_differences(row) = NaN; % If no positive values, assign NaN

end

end

% Display the array of smallest positive values from each row

disp('Array of smallest positive values from each row:');

disp(smallest_positive_differences);

% Calculating the root mean square of the differences

rms_difference = sqrt(mean(smallest_positive_differences.^2));

% Displaying the root mean square difference

fprintf('Root Mean Square Difference between the sets of dates: %.4f\n', rms_difference);

% Perform Monte Carlo simulation to assess statistical significance

num_iterations = 1000000; % Number of Monte Carlo iterations

randomised_rms = zeros(1, num_iterations);

for n = 1:num_iterations

% Generate X unique random indices within the range of the larger array

% (where X is the same as the number of observed NAO events)

random_indices = randperm(study_window_length, length(nao_years));

% Use random indices to select corresponding dates from the study window

rdm_selected_dates = study_window(random_indices);

% Calculate RMS difference between the datasets

% Create another matrix to store the results of volc years - randomly generated years

random_differences_matrix = zeros(length(vol_forcing_years), length(rdm_selected_dates));

% Perform subtraction and store the results in the matrix

for i = 1:length(vol_forcing_years)

for j = 1:length(rdm_selected_dates)

random_differences_matrix(i, j) = rdm_selected_dates(j) - vol_forcing_years(i);

end

end

% Find the smallest positive value in each column of the matrix

%Create an empty array to store the smallest positive value in each column

RANDOM_smallest_pos_diff = zeros(1, size(random_differences_matrix, 1));

for row = 1:size(random_differences_matrix, 1)

row_values = random_differences_matrix(row, :); % Extract column values

positive_values = row_values(row_values >= 0); % Filter positive values

if ~isempty(positive_values)

RANDOM_smallest_pos_diff(row) = min(positive_values); % Find the smallest positive value

else

RANDOM_smallest_pos_diff(row) = NaN; % If no positive values, assign NaN

end

end

rand_rms = sqrt(mean(RANDOM_smallest_pos_diff.^2));

randomised_rms(n) = rand_rms;

end

%plot histogram of MCS results

histogram(randomised_rms) %couldnt get BinWidth command to work

line([rms_difference, rms_difference], ylim, 'Color', 'r', 'LineWidth', 1.5)

%xlim([0 80])

xlabel('RMS Statistic');

ylabel('Frequency');

title('Monte Carlo Simulation of RMS Differences');

legend('Simulated RMS values', 'Observed RMS value', 'Location', 'best');

%calculate p-value

%rms_difference = abs(rms_data1 - rms_data2);

p_value = sum(randomised_rms <= rms_difference) / num_iterations;

% Display results

disp(['Calculated p-value: ', num2str(p_value)]);

**Appendix 2** – Additional simulated datasets:

*Top 20 NH points determined years (in order):*

[1902, 1991, 1912, 1933, 1980, 1956, 1982, 1907, 1986, 2008, 1981, 2020, 1924, 2011, 1931, 1966, 2013, 2010, 2015, 1929]

*Top 20 SH points determined years (in order):*

[1932, 1963, 1991, 2011, 2021, 2014, 1951, 1919, 2010, 2017, 1955, 2002, 1966, 1911, 1937, 1968, 2000, 1904, 1933, 2006]

*Top 12 NH points determined years pre-1950 (in order):*

[1902, 1912, 1933, 1907, 1924, 1931, 1929, 1917, 1947, 1914, 1932, 1913]

*Top 12 NH points determined years post-1950 (in order):*

[1991, 1980, 1956, 1982, 1986, 2008, 1981, 2020, 2011, 2013, 2010, 2015]

Historical El Niño events:

[1903, 1906, 1915, 1919, 1926, 1931, 1941, 1942, 1958, 1966, 1973, 1978, 1980, 1983, 1987, 1988,1992, 1995, 1998, 2003, 2007, 2010, 2016]
